# Evaluations of Healthcare Providers’ Perceived Support From Personal, Hospital, and System Resources: Implications for Well-Being and Management in Healthcare in Montreal, Quebec, During COVID-19

**DOI:** 10.1177/01632787211012742

**Published:** 2021-04-27

**Authors:** Nigel Mantou Lou, Tina Montreuil, Liane S. Feldman, Gerald M. Fried, Mélanie Lavoie-Tremblay, Farhan Bhanji, Heather Kennedy, Pepa Kaneva, Susan Drouin, Jason M. Harley

**Affiliations:** 1 Research Institute of the McGill University Health Centre, Montreal, Quebec, Canada; 2 Department of Educational and Counselling Psychology, 5620 McGill University, Montreal, Quebec, Canada; 3 Department of Psychiatry, 5620 McGill University, Montreal, Quebec, Canada; 4 Department of Surgery, Faculty of Medicine and Health Sciences, 5620 McGill University, Montreal, Quebec, Canada; 5 The Steinberg-Bernstein Centre for Minimally Invasive Surgery, 5620 McGill University, Montreal, Quebec, Canada; 6 Steinberg Centre for Simulation and Interactive Learning, 5620 McGill University, Montreal, Quebec, Canada; 7 Institute for Health Sciences Education, Montreal, Quebec, Canada; 8 Department of Pediatrics, Faculty of Medicine and Health Sciences, 5620 McGill University, Montreal, Quebec, Canada; 9 Ingram School of Nursing, 5620 McGill University, Montreal, Quebec, Canada

**Keywords:** healthcare provider, support, stress, resources, COVID-19

## Abstract

Increased stressful experiences are pervasive among healthcare providers (HCPs) during the COVID-19 pandemic. Identifying resources that help mitigate stress is critical to maintaining HCPs’ well-being. However, to our knowledge, no instrument has systematically examined how different levels of resources help HCPs cope with stress during COVID-19. This cross-sectional study involved 119 HCPs (64 nurses and 55 physicians) and evaluated the perceived availability, utilization, and helpfulness of a list of personal, hospital, and healthcare system resources. Participants also reported on their level of burnout, psychological distress, and intentions to quit. Results revealed that HCPs perceived the most useful *personal resource* to be family support; the most useful *hospital resources* were a safe environment, personal protective equipment, and support from colleagues; the most useful *system resources* were job protection, and clear communication and information about COVID. Moreover, HCPs who perceived having more *available hospital resources* also reported lower levels of psychological distress symptoms, burnout, and intentions to quit. Finally, although training and counseling services were perceived as useful to reduce stress, training was not perceived as widely available, and counseling services, though reported as being available, were underutilized. This instrument helps identify resources that support HCPs, providing implications for healthcare management.

## Background

Increased stressful experiences and psychological distress are prevalent among healthcare providers (HCPs) during the COVID-19 pandemic (Krishnamoorthy et al., 2020; Lou et al., 2021; Pappa et al., 2020). Identifying resources to mitigate stress, maintain HCPs’ well-being and prevent long-lasting negative impacts is critical (Dzau et al., 2020; Shapiro & McDonald, 2020). In this study, we developed a list of resources of potential relevance based on previous research to understand HCPs’ needs during COVID-19. We addressed three questions: (a) What are the most useful resources for nurses and physicians to cope with stress during the COVID-19 pandemic? (b) What resources are underutilized or lacking in the hospital and healthcare system for HCPs to cope with stress? (c) Does the amount of available personal, hospital, and healthcare system resources predict HCPs’ psychological distress, burnout, and intentions to quit?

## Method

### Participants

Data were collected from July 31st to August 15th 2020, at a University-affiliated tertiary care hospital network in Quebec, Canada, where there are approximately 1,350 physicians and 3,200 nurses. The study was approved by the [institutional] research ethics board. The survey invitation was sent out to a nurse manager in charge of the nursing administration and 80 unit heads to forward to nurses and physicians. We received 153 responses (3.4% of the HCPs in the hospital network).

### Measures

#### Personal, hospital, and healthcare system resources

Participants were provided with three lists of supports and resources (personal, hospital, and healthcare system resources) that may help them manage their stress (see Online Supplemental Material). The lists of resources were compiled from previous studies (Von Rueden et al., 2010), including studies about HCPs’ perceived support during SARS (Chan & Huak, 2004; Fiksenbaum et al., 2007; Lee et al., 2005), and research team members who are experienced physicians and nurses. Participants rated whether each support/resource was (a) not “available to me,” (b) “available, and I use it,” and (c) “available, but I do not use it.” Further, participants who indicated that a resource was available (i.e., option b or c) evaluated whether that resource helped them/ would help them.

#### Depression, Anxiety, and Stress Scale

Participants’ responded to a validated Depression, Anxiety, and Stress Scale (21-item; Henry & Crawford, 2005; Talaee et al., 2020) on a 4-point scale (“0 = did not apply to me at all” to “3 = very much or most of the time”). The total scores were calculated (α = .94).

#### Maslach Burnout Inventory

Participants responded to the Maslach Burnout Inventory for Medical Personnel (nine item; Riley et al., 2018) on a 7-point scale from “never” to “every day.” The mean scores were calculated (α = .79).

#### Intentions to quit

Participants responded to two items asking them whether they were thinking about leaving their employment and/or their profession (O’Driscoll & Beehr, 1994).

### Analyses

Descriptive analyses were conducted to examine the percentages of personal, hospital, and healthcare system resources that were perceived as available, utilized, and helpful among nurses and physicians, respectively. The total numbers of available personal, hospital, and system resources were calculated for each participant. Three multiple linear regression models were conducted to examine whether the numbers of available resources were associated with psychological distress, burnout, and intentions to quit, respectively.

## Results

The final sample (*n* = 119) included 64 nurses (58 females) and 55 physicians (30 females) after excluding participants who did not complete the survey or provide information regarding their position. A sensitivity analysis revealed that *n* = 119 was sufficient to detect a small effect size (*f*^2^ = .09). However, we did not have enough statistical power to examine differences among HCPs from different units (see Table S3 in the Online Supplemental Material) nor individual differences, including minority status (36 were racial and seven sexual orientation minorities).

### Personal Resources (see Online Supplemental Material for Details)

Most adaptive personal resources listed (family support, support from friends, hobbies, comfort food, and spending time in nature) were reported as widely available (>89%). Among the available personal resources, both nurses and physicians reported that they spent time in nature (86%, 71%), relied on family support (78%, 91%) and hobbies (76%, 81%), respectively. The most *helpful* personal resources available to nurses and physicians were family support (90%, 96%), support from friends (90%, 81%), and spending time in nature (98%, 92%).

### Hospital Resources

HCPs reported that personal protective equipment was widely available (≥94%), psychological counseling was perceived to only be available to 63% of the nurses and 57% of the physicians. The most useful hospital resource was also personal protective equipment. Most HCPs (95% of nurses and 86% of physicians) also perceived support from colleagues as very useful. Notably, most HCPs (93% nurses and 81% physicians), who received skills training and resilience training, rated the training as helpful, but most HCPs thought training was not available. This finding suggests that there was a large, reported gap between needs and resources related to training. Regarding underutilized hospital resources, counseling services were perceived as available to more than half of the HCPs and as helpful to most of the HCPs (88% nurses and 77% physicians), but only around 10% of them used this resource.

### System Resources

Resources that were perceived to be less available to HCPs were rewards/incentives (59% nurses and 15% physicians) and job protection (46% nurses and 15% physicians). The most useful (reported) healthcare system resources were job protection, clear communication and disease information, and continued implementation of social distancing. Although job protection was perceived as helpful, most HCPs reported that it was not available.

### Available Resources and Psychological Outcomes

The regression results are presented in Table 1 (see Online Supplemental Material for correlations). Regarding psychological distress, the availability of hospital resources (but not personal or healthcare system resources) significantly predicted reduced psychological distress. Similarly, the availability of hospital resources (but not personal and healthcare system resources) significantly and negatively predicted intentions to quit.

**Table 1.** Linear Regression of the Numbers of Resources on Psychological Distress, Burnout, and Intentions to Quit.

	*R* ^2^	*b*	*SE*	β	*t*	*p*	95% CI
Psychological distress (DASS)	.07*						
Personal resources		0.95	0.72	.12	1.33	.186	[−0.464, 2.368]
Hospital resources		−0.76	0.28	−.30	−2.77**	.006	[−1.308, −0.218]
Healthcare system resources		0.56	1.02	.06	−0.55	.584	[−1.460, 2.578]
Burnout	.03						
Personal resources		.002	0.07	.003	0.03	.974	[−0.128, 0.132]
Hospital resources		−0.05	0.03	−.20	−1.78	.078	[−0.095, 0.005]
Healthcare system resources		0.02	0.09	.02	0.22	.825	[−0.164, 0.206]
Intentions to quit	.11**						
Personal resources		−.001	0.03	−.004	−0.05	.962	[−0.060, 0.057]
Hospital resources		−0.04	0.01	−.38	−3.59***	<.001	[−0.063, −0.018]
Healthcare system resources		0.07	0.04	.18	1.75	.081	[−0.009, 0.157]

*Note.* No multicollinearity was detected (VIFs ≤ 1.45).

***p < .001. **p < .01. *p < .05.

## Discussion

This study identified resources that support HCPs and have implications for healthcare management. First, HCPs found the most useful resources for stress management were hospital, not personal resources (see also Panagioti et al., 2017). HCPs who reported more support from *hospital resources* experienced fewer psychological distress symptoms and were less likely to quit. However, managers need to consider the resources that are available, underutilized *and* considered useful when developing effective strategies for stress management as even those rated as available and useful were underutilized, particularly by physicians. HCPs reported that support from colleagues was one of the most helpful institutional resources, indicating a need for peer-support programs given HCPs’ willingness to receive collegial support (Hu et al., 2012).

### Limitation

The list of resources was based on previous studies and may not cover all possible resources. Because the measures were self-reports, there may be differences between what resources (e.g., counseling) were available and perceived as available. The sample represents a relatively small number of HCPs working in the hospital network and may not be a representative one. It is possible that HCPs who were experiencing greater levels of stress were more motivated to take part in the survey. Like other studies about HCPs’ experiences during COVID in a single hospital network (e.g., *n* = 96 in Beneria et al., 2020; *n* = 127 in Rubin et al., 2021), our sample was constrained by HCPs’ availability.

### Conclusion

HCPs’ stress and burnout are ongoing issues in the healthcare system (Spinelli et al., 2016; Thrush et al., 2021; Wang et al., 2019), which has been further impacted by the pandemic. Our findings show that HCPs are facing mental health challenges during the pandemic, and hospital resources are critical to protecting their well-being.

## Supplemental Material

Supplemental Material, sj-docx-1-ehp-10.1177_01632787211012742 - Evaluations of Healthcare Providers’ Perceived Support From Personal, Hospital, and System Resources: Implications for Well-Being and Management in Healthcare in Montreal, Quebec, During COVID-19 Click here for additional data file. Supplemental Material, sj-docx-1-ehp-10.1177_01632787211012742 for Evaluations of Healthcare Providers’ Perceived Support From Personal, Hospital, and System Resources: Implications for Well-Being and Management in Healthcare in Montreal, Quebec, During COVID-19 by Nigel Mantou Lou, Tina Montreuil, Liane S. Feldman, Gerald M. Fried, Mélanie Lavoie-Tremblay, Farhan Bhanji, Heather Kennedy, Pepa Kaneva, Susan Drouin and Jason M. Harley in Evaluation & the Health Professions
