# Supplementary material for: Evaluations of Healthcare Providers’ Perceived Support From Personal, Hospital, and System Resources: Implications for Well-Being and Management in Healthcare in Montreal, Quebec, During COVID-19
Source: Eval Health Prof. 2021 Apr 27;44(3):319–22. doi: 10.1177/01632787211012742 (PMC8326888; doi:10.1177/01632787211012742)
Supplement: Supplemental Material, sj-docx-1-ehp-10.1177_01632787211012742 - Evaluations of Healthcare Providers’ Perceived Support From Personal, Hospital, and System Resources: Implications for Well-Being and Management in Healthcare in Montreal, Quebec, During COVID-19 [file sj-docx-1-ehp-10.1177_01632787211012742.docx]

**Online Appendix Supplement for:**

Notes from the Field: Evaluations of healthcare providers’ perceived support from personal, hospital, and system resources: Implications for well-being and management in healthcare in Montreal, Quebec during COVID-19

**Appendix. Table S1**

*Perceived availability, utilization, and helpfulness of personal, hospital, and system resources to cope with stress*

|  |  | Nurses (*n* = 64) | | |  | | Physicians (*n* = 55) | | |
| --- | --- | --- | --- | --- | --- | --- | --- | --- | --- |
|  |  | Available | If Available | | |  | Available | If Available | |
|  | |  | Use | Helpful | |  |  | Use | Helpful |
| ***Personal Resources*** | |  |  |  | |  |  |  |  |
|  | Substance use | 21 (33%) | 4 (19%) | 8 (40%) | |  | 15 (27%) | 2 (13%) | 1 (7%) |
|  | Spiritual or religious | 44 (69%) | 19 (43%) | 28 (65%) | |  | 41 (75%) | 14 (34%) | 23 (56%) |
|  | Alcohol use | 51 (80%) | 23 (45%) | 22 (44%) | |  | 48 (89%) | 15 (31%) | 12 (25%) |
|  | Talking to psychologist | 52 (81%) | 17 (33%) | 41 (80%) | |  | 45 (82%) | 16 (36%) | 30 (67%) |
|  | Spending time in nature | 58 (91%) | 50 (86%) | 55 **(98%)** | |  | 49 (89%) | 35 (71%) | 45 (**92%**) |
|  | Family support | 59 (94%) | 46 (78%) | 52 (**90%**) | |  | 47 (85%) | 43 (91%) | 45 (**96%**) |
|  | My hobbies | 59 (94%) | 45 (76%) | 58 **(100%)** | |  | 52 (95%) | 42 (81%) | 26 (51%) |
|  | Comfort food | 60 (94%) | 45 (75%) | 37 (63%) | |  | 51 (93%) | 34 (67%) | 25 (49%) |
|  | Support from friends | 62 (97%) | 44 (71%) | 55 **(90%)** | |  | 52 (95%) | 37 (71%) | 42 (**81%**) |
| ***Hospital Resources*** | |  |  |  | |  |  |  |  |
|  | Reduced workload | 14 **(22%**) | 8 (57%) | 12 (**92%)** | |  | 8 (15%) | 6 (75%) | 7 (**70%**) |
|  | Family care (e.g., for children, sick family members) | 16 (25%) | 4 (25%) | 11 (73%) | |  | 14 (26%) | 8 (57%) | 10 (71%) |
|  | Appropriate pay | 21 (**33%**) | 18 (86%) | 19 (**90%)** | |  | 28 (51%) | 26 (93%) | 27 (96%) |
|  | Transportation | 24 (38%) | 17 (71%) | 18 (75%) | |  | 11 (20%) | 7 (64%) | 9 (90%) |
|  | Personal support to enable HCPs to perform their duties | 27 (43%) | 13 (48%) | 20 (77%) | |  | 15 (27%) | 7 (47%) | 13 (87%) |
|  | Skills training for expected duties and resilience training | 28 (**44%**) | 21 (75%) | 25 (**93%)** | |  | 15 **(27%)** | 10 (67%) | 13 (**81%)** |
|  | Opportunity to give feedback to hospital management | 33 (52%) | 15 (45%) | 22 (69%) | |  | 27 (49%) | 7 (26%) | 16 (59%) |
|  | Nap room | 37 (59%) | 27 (73%) | 28 (78%) | |  | 3 (05%) | 0 (0%) | 3 (1%) |
|  | Ability to influence clinical decisions | 38 (59%) | 29 (76%) | 33 (87%) | |  | 36 (65%) | 32 (89%) | 34 (94%) |
|  | Psychological counselling | 40 (63%) | 5 (13%) | 35 (**88%**) | |  | 31 (57%) | 3 (10%) | 23 (**77%**) |
|  | Communication on what is expected | 42 (68%) | 21 (50%) | 34 (83%) | |  | 32 (58%) | 24 (75%) | 24 (75%) |
|  | Food | 42 (67%) | 32 (76%) | 37 (93%) | |  | 30 (55%) | 23 (77%) | 19 (68%) |
|  | Support from hospital administration | 43 (67%) | 6 (14%) | 27 (64%) | |  | 25 (45%) | 4 (16%) | 15 (60%) |
|  | A safe environment | 44 (69%) | 38 (86%) | 39 (**95%**) | |  | 48 (87%) | 46 (96%) | 45 (**96%**) |
|  | Medical advice, e.g., screening when reporting for duty | 44 (69%) | 34 (77%) | 33 (79%) | |  | 45 (82%) | 37 (82%) | 37 (84%) |
|  | communication on how to minimize personal risk | 50 (78%) | 33 (66%) | 40 (83%) | |  | 40 (73%) | 35 (88%) | 30 (75%) |
|  | Support from your supervisor/manager/head | 51 (80%) | 17 (33%) | 36 (71%) | |  | 31 (56%) | 7 (23%) | 18 (58%) |
|  | Precautionary measures taken at work | 55 (86%) | 48 (87%) | 49 (92%) | |  | 48 (87%) | 47 (98%) | 45 (94%) |
|  | Support from your colleagues | **60 (95%)** | **43 (72%)** | **56 (95%)** | |  | **44 (80%)** | **32 (73%)** | **38 (86%)** |
|  | Resources (e.g., personal protective equipment) | 60 (**94%)** | 55 (92%) | 58 (**98%**) | |  | 52 (**96%)** | 50 (96%) | 50 (**98%**) |
| ***System resources*** | |  |  |  | |  |  |  |  |
|  | Job protection during the pandemic | 29 (**46%)** | 11 (38%) | 24 (**92%**) | |  | 8 (**15%**) | 8 (100%) | 8 (**100%**) |
|  | Rewards and incentive | 38 (59%) | 26 (68%) | 28 (76%) | |  | 8 (15%) | 5 (63%) | 5 (63%) |
|  | Free counselling and psychological support programs | 40 (**63%**) | 8 (**20%**) | 35 (**92%**) | |  | 24 (**44%**) | 1 (**4%)** | 12 (**50%**) |
|  | Clear communication and disease information about COVID-19 | 57 (90%) | 47 (82%) | 52 (**95%**) | |  | 48 (87%) | 44 (92%) | 45 (**94%**) |
|  | Continued implementation of social distancing to reduce the spread of the COVID-19 | 61 (95%) | 55 (90%) | 56 (**97%**) | |  | 52 (95%) | 51 (98%) | 49 (**94%**) |

**Appendix. Table S2**

*Correlation between available resources and psychological distress, burnout, and intentions to quit.*

|  | 1 | 2 | 3 | 4 | 5 | 6 |
| --- | --- | --- | --- | --- | --- | --- |
| 1.Personal Resources | -- |  |  |  |  |  |
| 2.Hospital Resources | .26^**^ | -- |  |  |  |  |
| 3.System Resources | .08 | **.51^***^** | -- |  |  |  |
| 4.Psychological Distress | .05 | **-.24^**^** | -.09 | -- |  |  |
| 5.Burnout | -.05 | **-.18^*^** | -.08 | **.63^***^** | -- |  |
| 6.Intentions to Quit | -.09 | **-.29^**^** | -.01 | **.28^**^** | **.46^***^** | -- |
| *M* | 7.28 | 12.41 | 3.07 | 14.59 | 2.84 | 0.36 |
| *SD* | 1.51 | 4.54 | 1.18 | 11.54 | 1.04 | 0.48 |

*Note.* ^***^*p* < .001, ^**^*p* < .01, ^*^*p* < .05. The results of Pearson correlations showed that the numbers of available *personal resources* were not statistically significantly correlated with DASS, burnout, or intentions to quit. Second, the number of *available hospital resources* was significantly correlated with DASS, burnout, and intention to quit. Finally, the number of *healthcare system resources* was not significantly correlated with DASS, burnout, or intention to quit.

**Appendix Table S3**

Participants Unit information

| Unit | Nurses | Physicians |
| --- | --- | --- |
| Emergency | 3 (4.7%) | 24 (43.6%) |
| Intensive care | 2 (3.1%) | 14 (25.5%) |
| Long-term care | 0 | 3 (5.5%) |
| Surgical | 27 (42.2%) | 33 (60%) |
| Mentalh health | 1 (1.6%) | 4 (7.3%) |
| Obstetrics | 0 | 7 (12.7%) |
| Operating/Recovery | 23 (35.9%) | 10 (18.2%) |
| Pediatrics | 1 (1.6%) | 10 (18.2%) |
| Other | 6 (9.4%) | 7 (12.7%) |

*Note.* Some HCPs worked in more than one unit.
